# Prediction Models for Complete Resection in Secondary Cytoreductive Surgery of Patients With Recurrent Ovarian Cancer

**DOI:** 10.3389/fonc.2021.674637

**Published:** 2021-09-23

**Authors:** Caixia Jiang, Zhengyu Li

**Affiliations:** Department of Obstetrics and Gynecology, Key Laboratory of Birth Defects and Related Diseases of Women and Children, Ministry of Education, West China Second University Hospital, Sichuan University, Chengdu, China

**Keywords:** complete gross resection, recurrent ovarian cancer (ROC), MSK criteria, AGO score, Tian model, secondary cytoreductive surgery (SCS)

## Abstract

The most advanced epithelial ovarian cancer develops recurrent disease despite maximal surgical cytoreduction and adjuvant platinum-based chemotherapy. Treatment with secondary cytoreductive surgery (SCS) combined with chemotherapy or with chemotherapy alone for patients with platinum-sensitive recurrent ovarian cancer (ROC) is currently under heated discussion. Encouragingly, the results of the AGO DESKTOP III Study and the SOC1/SGOG-OV2 trial, which have been published recently, showed a striking advantage in terms of overall survival (OS) and progression-free survival (PFS) of ROC patients undergoing SCS compared to chemotherapy alone; moreover, a benefit of SCS exclusively for patients with complete gross resection (CGR) was particularly highlighted. CGR is considered the ultimate goal of SCS, on condition that the balance between maximal survival gain and minimal operative morbidity is maintained. Several models have been proposed to predict the rate of CGR, such as the MSK criteria, the AGO score, and the Tian model, over the last 15 years. This summary is mainly about the several previously published prediction models for CGR in SCS of ROC patients and discusses the effectiveness and limitations of these prediction models.

## Introduction

### The Vital Significance of CGR in SCS

Ovarian cancer is the leading cause of death and the second most common gynecological cancer (1). Primary debulking surgery followed by platinum-based chemotherapy with or without first-line maintenance therapy with bevacizumab or emerging targeted drugs remains the standard treatment of advanced epithelial ovarian cancer (2). Despite the fact that 80% of patients attain clinical complete remission *via* initial therapy, unfortunately, about 80% of patients can relapse within 3 years, including platinum-resistant and platinum-sensitive recurrence. The average 5-year survival rate following recurrence is less than 10% (3). Surgery and medical treatment are the cornerstones of recurrent ovarian cancer (ROC) therapy. For patients with platinum-resistant ROC, secondary cytoreductive surgery (SCS) is usually not indicated due to the limited life expectancy and surgical morbidity/mortality, while patients with platinum-sensitive ROC can be treated with SCS combined with chemotherapy (platinum-based) or with chemotherapy alone (4). SCS is defined as an operation performed on patients who have either persistent disease at the completion of a planned course of chemotherapy or who subsequently experience clinical relapse, and the survival benefits of surgery need to be weighed against the risks of morbidity and mortality (5, 6). As for platinum-sensitive ROC, the role of SCS in ROC has so far not been fully confirmed by prospective randomized surgical trials, although SCS has been listed as an optional treatment in the National Comprehensive Cancer Network (NCCN) guidelines, which is primarily based on the results of a few single-center and multicenter retrospective case studies and limited meta-analyses (6–8).

In fact, the biggest limitation of these studies is the inherent patient selection bias, which is difficult to avoid in the absence of randomized clinical trials. Stirringly, the final results of the AGO DESKTOP III trial (NCT01166737) were announced in an oral presentation at the 2020 ASCO Annual Meeting (Abstract 6000). The results showed that patients undergoing SCS combined with chemotherapy benefited in terms of median overall survival (mOS = 53.7 *vs.* 46.0 months) and median progression-free survival (mPFS = 18.4 *vs.* 14.0 months) compared with those undergoing chemotherapy alone without increased surgical morbidity/mortality. More importantly, the study confirmed that complete gross resection (CGR) of macroscopic disease was the key point and that patients with any residual disease (even optimal) did not benefit from SCS (mOS = 61.9 *vs.* 28.8 months), even worse than those having chemotherapy alone (mOS = 61.9 *vs.* 46.0 months) (9). Simultaneously, the results of the SOC1/SGOG-OV2 trial (NCT01611766) were presented at the meeting (Abstract 6001) and subsequently published online by Zang et al. (10, 11). Compared with chemotherapy alone, both PFS (17.4 *vs.* 11.9 months) and the median time to start of the first subsequent therapy (TFST = 18.1 *vs.* 13.6 months) were in favor of the patients accepting SCS combined with chemotherapy. Moreover, the interim OS analysis showed that mOS was 58.1 months (95% CI not estimable to not estimable) in the surgery group and 53.9 (42.2–65.5) months in the no-surgery group (hazard ratio 0.82, 95% CI 0.57–1.19). Besides, the median accumulating treatment-free survival (TFSa) rates were 46.8 months in the surgery group and 42.4 months in the no-surgery group. Mature data on OS and TFSa are still awaited. Combining the previous subgroup analysis results of the GOG213 Study in 2019, it was shown that 150 patients with CGR after SCS, compared with those with residual tumor after surgery (89 patients), had longer OS (56.0 *vs.* 37.8 months) and longer PFS (22.4 *vs.* 13.1 months) (12, 13) (**Table 1**). In summary, all three randomized clinical trials (RCTs) showed a significant statistical advantage in PFS in the SCS group, with an even more significant difference in patients with CGR (about a 7-month increase in PFS). Data on OS are different in these two completed trials. With respect to the inconsistent results, a large amount of discussion focuses on issues such as platinum-free interval, pattern of recurrence, BRCA (breast cancer gene) status, and the use of bevacizumab and/or poly-ADP ribose polymerase (PARP) inhibitors (14, 15). Recently, a meta-analysis encompassed the above three RCTs and showed that SCS was superior to chemotherapy alone in terms of PFS, and particularly with PFS and OS benefits in the CGR subpopulation (8).

**Table 1:** Comparisons between the GOG213, AGO DESKTOP III, and SOC1/SGOG-OV2 trials.

		GOG213	AGO DESKTOP III	SOC1/SGOG-OV2
No. of patients		485	408	357
Year		2007–2011	2010–2014	2012–2019
Age (years)		57	60.5	54
Primary FIGO III–IV		86%	74.6%	82%
Selection criteria		CGR (individuation)	AGO score	iMODEL+ PET-CT
Histology (serous)		86%	85%	81%
Platinum-free interval (months)		19.7	19.9	16.1
CGR		67%	74.5%	76.7%
Mortality rate		30 days: 0.4%	90 days: 0.5%	60 days: 0%
Chemotherapy (platinum-based)		100%	89%	97%/96%
Bevacizumab (second-line)		84%	23%	1.1%
PARPi (second-line maintenance)		NA	3.9%	10.1%
Surgery *vs.* no surgery, *n* (HR, 95%CI)	mOS	50.6 *vs.* 64.7 (1.29, 0.97–1.72)	53.7 *vs.* 46.2 (0.76, 0.59–0.97)	58.1 *vs.* 53.9 (0.82, 0.57–1.19)^a^
	mPFS	18.9 *vs.* 16.2 (0.82, 0.66–1.01)	18.4 *vs.* 14.0 (0.66, 0.54–0.82)	17.4 *vs.* 11.9 (0.58, 0.45–0.74)
	TFST	NA	17.9 *vs.* 13.7 (0.65, 0.52–0.81)	18.1 *vs.* 13.6 (0.59, 0.46–0.76)
CGR *vs.* incomplete resection, *n* (HR, 95%CI)	mOS	56.0 *vs.* 37.8 (0.61, 0.40–0.93)	60.7 *vs.*28.8 (0.40, 0.28–0.59)	Pending
	mPFS	22.4 *vs.* 13.1 (0.51, 0.36–0.71)	21.2 *vs.* 13.7 (0.98, 0.71–1.35)	Pending
CGR *vs.* no surgery, *n* (HR, 95%CI)	mOS	56.0 *vs.* 64.7 (1.03, 0.74–1.46)	60.7 *vs.* 46.2 (0.57, 0.43–0.76)	Pending
	mPFS	22.4 *vs.* 16.2 (0.62, 0.48–0.80)	21.2 *vs.* 14.0 (0.56, 0.43–0.72)	Pending

FIGO, International Federation of Gynecology and Obstetrics; CGR, complete gross resection; PARPi, poly-ADP ribose polymerase inhibitor; HR, hazard ratio; TFST, time to start of the first subsequent therapy; NA, not applicable; mOS, median overall survival; mPFS, median progression-free survival.

a Results of the interim overall survival analysis.

Based on the three RCTs mentioned above, a point that draws our attention the most is that CGR has been robustly confirmed as the most crucial survival determinant in ROC. The ultimate goal of SCS should be the removal of all visible tumors. This is consistent with previous studies. A meta-analysis on the role of SCS for ROC reported that each 10% increase of complete resection rate translates into a 3-month increase of OS (16). In addition, various studies on SCS have shown that achieving CGR in SCS was the most vital factor associated with survival benefit (8, 17). Therefore, identifying valid prediction models for CGR in SCS is an urgent need, for two reasons: one is for the selection of patients most appropriate for surgery and the other is for avoiding surgical burden on the part of patients of both limited benefit from the procedure and limited overall life expectancy.

## Prediction Models for Proper Patient Selection to Achieve CGR in SCS

Almost all of the evidence indicated a benefit of SCS exclusively in patients with CGR. However, not every patient is suitable for complete resection surgery in consideration of the accompanying surgical morbidity and mortality rates. Over the last 15 years, several models have been developed for predicting surgical outcomes, PFS, or OS on the basis of the clinical and pathological data available at the primary diagnosis and recurrence (3). Among them, only the Memorial Sloan Kettering (MSK) criteria, the AGO (Arbeitsgemeinschaft Gynäkologische Onkologie) score, and the Tian model are the most often cited models with international validity, while others have not been externally verified. The models are introduced as follows.

### MSK Criteria

As early as 1998, the Second Ovarian Cancer Consensus Conference demonstrated the factors for the identification of optimal candidates for SCS: progression-free interval (PFI) >12 months, response to primary chemotherapy, good performance status, and feasible complete resection based on preoperative evaluation (3). Then, a large retrospective single-institution study of 153 patients (from 1987 to 2001) undergoing SCS was conducted by the MSK Cancer Center. This study suggested that the goal of SCS should be to achieve residual disease ≤0.5 cm. Then, a prediction model was established based on disease-free interval (DFI), the number of recurrence sites, and evidence of carcinomatosis with a CGR rate of 41% (**Table 2**) (18).

**Table 2:** MSK criteria.

Disease-free interval	Single site	Multiple sites: no carcinomatosis	Carcinomatosis^a^
6–12 months	Offer SCS	Consider SCS	No SCS
12–30 months	Offer SCS	Offer SCS	Consider SCS
>30 months	Offer SCS	Offer SCS	Offer SCS

MSK, Memorial Sloan Kettering; SCS, secondary cytoreductive surgery.

a Carcinomatosis was defined as the presence of 20 tumor nodules noted at the time of surgery.

### AGO Score

In about the same period as the MSK study, a series of AGO-DESKTOP OVAR trials on surgery in ROC were carried out. Firstly, a retrospective multicenter study (DESKTOP I trial) of 267 patients (from 2000 to 2003) found that CGR was associated with prolonged survival in ROC and developed a hypothesis for a predictive score to identify patients who had complete resection during SCS. Different from the MSK criteria, the AGO score consists of a good performance status, absence of ascites, and outcome of primary surgery/initial FIGO (International Federation of Gynecology and Obstetrics) stage (**Table 3**) (18). Secondly, the score model was subsequently verified in a multicenter trial (DESKTOP II trial) of 516 patients, which was the first prospectively validated study to positively predict surgical outcomes in ROC with a CGR rate of 76%. However, the negative predictive value was 38% and the specificity was low (53%), which could not be ignored either (19). Finally, the AGO DESKTOP III stood as a phase III prospective randomized controlled trial, as we have mentioned above—the AGO score was widely used in clinical practices (9).

**Table 3:** AGO score.

Predictive parameters of CGR
Platinum-sensitive ROC
Good performance status (ECOG 0)
No residual disease after primary surgery (or, alternatively, FIGO I/II)
Absence of ascites in preoperative imaging (<500 ml)

AGO, Arbeitsgemeinschaft Gynäkologische Onkologie; CGR, complete gross resection; ROC, recurrent ovarian cancer; FIGO, International Federation of Gynecology and Obstetrics; ECOG, Eastern Cooperative Oncology Group.

### Tian Model

To better assess the parameters associated with CGR in SCS, Tian et al. conducted a large retrospective multicenter international study on 1,075 patients (before 2009) with ROC undergoing SCS by collecting raw data from nine previously published studies including the MSK and AGO data. Besides, additional data on 117 patients (from 2007 to 2009) who were not included in the development of the model were used for external validation and to assess the discrimination of the model. CGR was achieved in 40% of the population, with rates ranging from 8.3% to 65.9%. After an analysis of the factors impacting the surgical outcomes of SCS, six significant parameters were identified *via* multivariate logistic regression, and each of them obtained a risk score based on the beta coefficient. According to the sum of the risk scores, patients would be categorized into the low-risk group (≤4.7) and the high-risk group (>4.7). The proportion of CGR in the low-risk group was 53.4%, while that in the high-risk group was 20.1% (**Table 4**). External validation of the Tian model showed sensitivity and specificity values of 83.3% and 57.6%, respectively. The area under the receiver operating characteristic curve for predicting CGR was 0.68 (20, 21).

**Table 4:** Tian model.

Impact factors	Scoring					
	0	0.8	1.5	1.8	2.4	3.0
FIGO stage	I/II	III/IV				
RD after primary surgery	0		>0			
PFI (months)	≥16				<16	
ECOG performance status	0–1				2–3	
CA_125_ at recurrence (U/ml)	≤105			>105		
Ascites at recurrence	Absent					Present

FIGO, International Federation of Gynecology and Obstetrics; ECOG, Eastern Cooperative Oncology Group; RD, residual disease; PFI, progression-free interval.

### Other Prediction Models

Due to the accumulated data confirming that CGR during SCS is associated with the largest survival benefit, whereas surgery with large tumor bulks of 1 cm diameter or more left does not alter the prognosis significantly, relevant studies have focused on the search for a prediction model for CGR to select the appropriate patients.

A single-center retrospective study analyzed 135 patients (from 2009 to 2013) with ROC and came up with an equation that allowed calculation of the SeC-score value. This study found with internal validation that the preoperative variables such as CA_125_, HE_4_, ascites, and residual disease (RD) at primary surgery were all involved in the risk of optimal SCS, with sensitivity and specificity of 82% and 83%, respectively (22).

A similar single-center retrospective study analyzed 80 patients (from 1982 to 2012) with ROC undergoing SCS using the grouping model. A total of four favorable prognostic factors were independently associated with better survival: treatment-free interval (TFI) >12 months, absent distant metastasis, solitary disease, and performance status (PS) = 0. Patients with three to four of these factors had better survival and higher CGR rates (79% *vs*. 40% *vs*. 33%) than those with two or none or only one factor. Therefore, SCS for patients with three to four of the above favorable factors at ROC was strongly recommended. SCS may be considered in patients with two factors (the Minaguchi criteria) if CGR is expected to be achieved, although prospective studies were warranted to validate the results (23, 24).

A few studies have conducted some exploration to select suitable patients with ROC for successful SCS by laparoscopy. Fanfani et al. (25) reported that this could be effective for the evaluation of candidates for CGR using PET-CT and a staging laparoscopy (S-LPS)-based method. This method had been validated with an overall accuracy rate for primary debulking ranging between 77.3% and 100%. At a total predictive index value (PIV) ≥8, the probability of optimal primary resection at laparotomy was equal to 0, and the rate of unnecessary exploratory laparotomy was 40.5% (26, 27). However, the subjective evaluation of PET-CT images and S-LPS in this study rather than a scoring standard limited its application and promotion. A similar limitation existed in the study of Yang et al. (28). The selection criteria were developed using a laparoscopic-based PIV score combined with assessment of the multidisciplinary team (MDT), but lacked quantification of the MDT.

Bogani et al. (29) reported an innovative method using artificial intelligence (AI), which was useful in weighing the importance of the clinical variables predicting CGR. As a result, three main factors—DFI (importance = 0.231), retroperitoneal recurrence (importance = 0.178), and RD at primary surgical treatment (importance = 0.138), were proposed to predict CGR using artificial neuronal network (ANN) analysis. However, these predictors have not yet been modeled and lack validity.

## Differences Among the Three Prediction Models (MSK, AGO, and Tian Model)

As mentioned above, only the MSK criteria, AGO score, and the Tian model have been externally validated in clinical studies. This review focuses on discussing the strengths and limitations of these main prediction models. In terms of the number of populations included in these studies, retrospective case data in the MSK criteria were limited by a single institution, while it was more comprehensive in the Tian model, with case data from a larger international multicenter. With respect to variables, the three models have a common point: that PFI or platinum-sensitive recurrent is considered as the most important predictive factor, without doubt, which shows a positive correlation with complete resection. Unlike the AGO score and the Tian model, the number of recurrence sites and peritoneal carcinomatosis are considered as negative predictors in the MSK criteria. This was confirmed by the DESKTOP I trial, demonstrating that patients with and without peritoneal carcinomatosis had complete resection rates of 26% and 74%, respectively (*p* < 0.0001). Peritoneal carcinomatosis was a negative predictor for complete resection, but had no effect on prognosis if complete resection is achieved. In the case of complete resection of peritoneal carcinomatosis, there was no difference in OS when compared with complete resection without peritoneal carcinomatosis (30). Another study also confirmed this viewpoint: that patients who have multisite recurrence tend to have shorter PFS, but that there is no difference in OS (31). In the development of the AGO score, peritoneal carcinomatosis and CA_125_ in preoperative diagnostics were not included in multivariate analysis because of their correlation with ascites; stepwise analysis with elimination of one of these three variables showed ascites being the most useful one (32). Based on this, we were inclined to think that the Tian model is quite similar to the AGO score, with only one additional factor, CA_125_, in the Tian model compared to the AGO score (21).

## Evaluation and External Verification of the Three Prediction Models

Recently, as valid selection criteria, both the AGO score and the Tian model have been prospectively validated in the form of increased PFS in DESKTOP III and SOC1/SGOG-OV2, respectively, while the MSK criteria lacked prospective validation up until now (9, 11). In the last decade, a numbers of retrospective studies demonstrated that the three prediction models (the MSK criteria, AGO score, and the Tian model) were widely applied in clinical practice to help inform decision-making for ROC patients. Harter et al. (33) performed an exploratory analysis to evaluate the decision effectiveness of the AGO score in 217 patients with SCS in ROC from 1999 to 2013, before and after introduction of the AGO score. The results showed that the AGO score could identify suitable candidates for SCS, with CGR being 89.3% and 66.7% in positive and negative AGO scores, respectively, indicating that the AGO score did not present a very good negative predictive value. The authors held the view that the selection criteria for surgery in patients with negative AGO score were not standardized, owing to the time span of the study. Nevertheless, it should be noted that 38% of the patients with a negative AGO score achieved absent residual tumor after SCS and that the PFS was comparable with that of patients with a positive AGO score. This aspect showed that the AGO score does not affect a patient’s inoperability. Therefore, further studies should be carried out to evaluate the predictive and prognostic impact of a negative score (4). Cowan et al. (34) conducted a population-based retrospective study of the MSK Cancer Center to compare the predictive value of the MSK with that of the validated Tian model and the AGO score. The results showed good concordance between the Tian model and MSK, with accuracy rates of 88% and 86%, respectively, in predicting CGR, while that of the AGO score was 49%. In addition, the MSK criteria were more user-friendly because fewer variables were involved. The Tian criteria may be applied to intermediate MSK cases for further stratification. The AGO score and the MSK criteria were retrospectively applied to 194 patients in another study to assess the probability of achieving CGR in SCS. The results showed that both models contributed to identifying patients undergoing SCS, while 63.4% of patients with a negative AGO score achieved CGR. Moreover, the concordance indices of two separate nomograms based on the AGO score and the MSK criteria (C-index values of 0.5900 and 0.5989, respectively) were also not high. Therefore, the authors implied that these models might be too strict that they exclude patients from the chance of a successful ROC surgery (35). Besides, several retrospective studies have argued that the AGO score and the Tian model show high positive predictive values for complete SCS, 80.0%–84.3% and 73%–80.3%, respectively, but also relatively high false negative rates of 61%–68.5% and 55.6%–70%, respectively. We would still highlight that both scores identify a subset of patients who could achieve CGR, but do not select patients who are suitable candidates for surgery compared to chemotherapy. Further studies and discussion are warranted so as not to prohibit patients from having potential life-extending surgery (36–38). In addition, preoperative imaging is an essential tool in making the right decision (4). Several studies have suggested that additional refinement of the score, such as with whole-body MRI or PET-CT, is needed to exclude women from SCS. Overall, the selection criteria and potential beneficial subpopulation of CGR could be ultimately refined in future clinical practice (38).

## Limitations of the Current Prediction Models

In view of the similar CGR and OS achieved in the three foregoing phase III randomized clinical trials, this provides proof that the AGO score and the Tian model are validated scoring criteria used to select appropriate patients to achieve CGR. Not only are patient criteria important, but there are also several limitations that lead to an unfavorable influence on the accuracy of the prediction models. Firstly, none of the models incorporates the surgeon’s own surgical ability as an evaluation parameter on the condition of distinct surgical experience of SCS. Patients with the same prediction scores undergoing SCS performed by a gynecologic oncologist or a general gynecologist may obtain different CGR rates. Secondly, differences in the clinical resources have not been included in the models in terms of the comprehensive capabilities of the MDT teams, which vary in different hospitals. As is known, some surgical operations such as those requiring intestinal and urological skills are involved during the SCS process and usually entail cooperation with MDT teams. Thirdly, most of the models lack preoperative imaging diagnosis to exclude inoperable patients with distant metastasis, such as in the lung or brain. As a result, adequate preoperative evaluations with whole-body MRI or PET-CT are necessary. Fourthly, so far, except for the three mainstay prediction models, the predictive value of the others has not been externally validated. Fifthly, recently, several retrospective studies have evaluated the impact of biological features, such as the BRCA status and the use of PARP inhibitors, with some controversial results on the benefits of SCS (39). Fagotti et al. found that patients with *BRCA1*/*BRCA2* wild type benefited from SCS, while for patients with *BRCA1*/*BRCA2* mutations, the benefit was not as obvious. Subsequently, their further study demonstrated that SCS increases the TFST and post-recurrence survival in platinum-sensitive ROC patients with *BRCA* ^mut^ candidates for olaparib maintenance after platinum-based chemotherapy (40, 41). Conversely, another study showed a benefit of SCS irrespective of the *BRCA1*/*BRCA2* status among patients mostly not treated with PARP inhibitors (42). Besides, a phase II multicenter RCT (SGOG SOC-3 Study, NCT03983226) was conducted to answer the question of whether patients can benefit from a potential CGR combined with niraparib maintenance in platinum-sensitive secondary recurrent patients. The results are awaited (43). Further research should be conducted to investigate the benefits of SCS in relation to the molecular characteristics (*BRCA* or homologous recombination deficiency status) and the use of PARP inhibitors and/or bevacizumab and to identify individualized surgical strategies, accordingly optimizing the prediction model for CGR in SCS for ROC patients.

## Conclusion

Adequate selection of ROC patients for surgery is crucial due to the primary goal of SCS of achieving CGR. In view of the above discussion, each of the three mainstay models—the MSK criteria, the AGO score, and the Tian model—has its strengths and limitations. They can be efficiently applied to clinical practice to help inform decision-making for ROC patients, but with relatively high false-negative rates, while other models need to be externally validated. Further prospective randomized surgical studies are warranted to compare the prediction accuracy and the advantages and disadvantages of those models. To sum up, choosing the right patient, right clinic, and right surgeon may be the key point to achieve good outcomes from SCS. We await an enhanced prediction model that integrates detailed clinical data of patients (such as preoperative imaging, molecular characteristics, and the use of bevacizumab and/or PARP inhibitors), the surgeon’s surgical ability, and the capability of the MDT team for achieving maximum CGR in SCS for ROC patients.

## Author Contributions

CJ wrote the manuscript. ZL provided practical suggestions and critically revised the manuscript. All authors contributed to the article and approved the submitted version.

## Funding

This work was supported by the Sichuan Province Science and Technology Support Program (grant no. 2019YJ0072).

## Conflict of Interest

The authors declare that the research was conducted in the absence of any commercial or financial relationships that could be construed as a potential conflict of interest.

## Publisher’s Note

All claims expressed in this article are solely those of the authors and do not necessarily represent those of their affiliated organizations, or those of the publisher, the editors and the reviewers. Any product that may be evaluated in this article, or claim that may be made by its manufacturer, is not guaranteed or endorsed by the publisher.
